# Design guidelines for an electron diffractometer for structural chemistry and structural biology

**DOI:** 10.1107/S2059798319003942

**Published:** 2019-04-08

**Authors:** Jonas Heidler, Radosav Pantelic, Julian T. C. Wennmacher, Christian Zaubitzer, Ariane Fecteau-Lefebvre, Kenneth N. Goldie, Elisabeth Müller, Julian J. Holstein, Eric van Genderen, Sacha De Carlo, Tim Gruene

**Affiliations:** a Paul Scherrer Institut, 5232 Villigen PSI, Switzerland; b DECTRIS Ltd, 5405 Baden-Daettwil, Switzerland; c Scientific Center for Optical and Electron Microscopy, ETH Zürich, 8093 Zürich, Switzerland; d Center for Cellular Imaging and NanoAnalytics, University Basel, 4058 Basel, Switzerland; e Faculty of Chemistry and Chemical Biology, TU Dortmund University, Otto Hahn Strasse 6, 44227 Dortmund, Germany

**Keywords:** electron diffractometer, EIGER X 1M detector, 3D electron diffraction, chemical crystallography, EIGER hybrid pixel detector, structural chemistry

## Abstract

3D electron diffraction has reached a stage where the structures of chemical compounds can be solved productively. Instrumentation is lagging behind this development, and to date dedicated electron diffractometers for data collection based on the rotation method do not exist. Current studies use transmission electron microscopes as a workaround. These are optimized for imaging, which is not optimal for diffraction studies. The beam intensity is very high, it is difficult to create parallel beam illumination and the detectors used for imaging are of only limited use for diffraction studies. In this work, the combination of an EIGER hybrid pixel detector with a transmission electron microscope to construct a productive electron diffractometer is described. The construction not only refers to the combination of hardware but also to the calibration of the system, so that it provides rapid access to the experimental parameters that are necessary for processing diffraction data. Until fully integrated electron diffractometers become available, this describes a setup for productive and efficient operation in chemical crystallography.

## Abstract 2

Installation of the EIGER X 1M detector onto an electron microscope and system calibration for data collection turns a transmission electron microscope into an electron diffraction instrument. Data can be collected and processed with a throughput that meets the requirements of a modern X-ray facility. The setup described here offers access to single-crystal structure determination from microcrystalline powder when large single crystals cannot be produced.

## 1. Introduction   

We recently demonstrated that 3D electron diffraction has reached a productivity level comparable to X-ray structure determination in chemical crystallography (Gruene, Wennmacher *et al.*, 2018 ▸). This is only one of several recent publications that show how 3D electron diffraction is a fast-progressing, exciting research area (Simancas *et al.*, 2016 ▸; Palatinus *et al.*, 2017 ▸; Wang, Rhauderwiek *et al.*, 2018 ▸). Crystallography in general is one of the main technologies for determining the three-dimensional coordinates of atoms within molecules. It therefore serves as a key method for a wide range of applications in chemistry, materials science, biochemistry and many other fields. Structure-based design of inorganic and organic compounds is critical for modern energy storage, catalyst optimization and drug design (Ariëns, 1984 ▸; Brameld *et al.*, 2008 ▸; Mentzen, 2007 ▸; Parsons *et al.*, 2013 ▸; Dalle *et al.*, 2014 ▸; Gruene, Li *et al.*, 2018 ▸; Lee *et al.*, 2018 ▸; Wang, Rhauderwiek *et al.*, 2018 ▸). Data are generally collected using the rotation method: the crystal is rotated on an axis while being irradiated with a planar, usually monochromatic wave. The diffraction pattern is recorded contiguously with an area detector (Arndt & Wonacott, 1977 ▸; Pflugrath, 1999 ▸; Leslie, 2006 ▸). The vast majority of structures are determined with X-ray radiation, but neutrons and electrons are also available as radiation sources. The different physics of interaction reflects the different application of each type of radiation (Giacovazzo, 1985 ▸; Blakeley *et al.*, 2008 ▸; Zou *et al.*, 2011 ▸). Particularly in X-ray crystallography, modern instrumentation and advances in computing power enable data collection, data processing, structure solution and refinement within a couple of hours. The annual growth rates of the Cambridge Structural Database, the Crystallographic Open Database and the Protein Data Bank have reached 50 000, 18 000 and 10 000 new structures, respectively (Groom *et al.*, 2016 ▸; Merkys *et al.*, 2016 ▸; wwPDB consortium, 2018 ▸). For both laboratory X-ray diffractometers and synchrotrons, all experimental parameters are stored as metadata so that data processing can start at the click of a button.

The introduction of hybrid pixel detectors in crystallo­graphy led to the development of shutterless and continuous data collection (Broennimann *et al.*, 2006 ▸). The combination of the rotation method with advanced detector technology had a great impact not only on X-ray crystallography but also on electron crystallography. Until recently, 3D electron crystallography was mainly the domain of materials science. Nowadays, crystal structures can also be determined from radiation-sensitive compounds such as metal–organic frameworks, zeolites, organic and pharmaceutical compounds, and macromolecules (Kolb *et al.*, 2010 ▸; Gorelik *et al.*, 2012 ▸; Shi *et al.*, 2013 ▸; Yonekura *et al.*, 2015 ▸; Gemmi *et al.*, 2015 ▸; Simancas *et al.*, 2016 ▸; van Genderen *et al.*, 2016 ▸; Palatinus *et al.*, 2017 ▸; Clabbers *et al.*, 2017 ▸; Zhang *et al.*, 2018 ▸; Wang, Yang *et al.*, 2018 ▸; Gruene, Li *et al.*, 2018 ▸; Gruene, Wennmacher *et al.*, 2018 ▸). The size of single crystals for routine structure determination is limited to a side length of about 5–10 µm when X-rays are used as a radiation source. With electron radiation, no such size limit exists (Dimmeler *et al.*, 2000 ▸). Experiments in 3D electron crystallography are normally carried out with transmission electron microscopes (TEM) as the radiation source. These instruments are not designed for diffraction studies and thus have a number of shortcomings, many of which have been pointed out (Lanza *et al.*, 2019 ▸; Gemmi *et al.*, 2015 ▸). As a result, data collection and consequently data processing is complicated and time-consuming, and requires workaround solutions (Hattne *et al.*, 2015 ▸). Because of the small sample size of less than 1 µm and a beam size of between 2 µm and as low as 100 nm, the stability of the goniometer rotation must be greater than for X-ray diffractometers, as otherwise the crystal can move out of the beam upon rotation. TEMs are designed to provide high beam intensity, as required for imaging applications, while diffraction requires much lower intensity. A recent hardware improvement has been the introduction of hybrid pixel detectors for electron diffraction studies (Casanas *et al.*, 2016 ▸; van Genderen *et al.*, 2016 ▸; Tinti *et al.*, 2018 ▸). However, to date no smooth integration between such detectors and a TEM exists, and the experimental parameters required for data processing must be calibrated manually. The development of automated data collection is a first move in the right direction (Smeets *et al.*, 2018 ▸; Cichocka *et al.*, 2018 ▸). Modern data-integration programs such as *DIALS*, *XDS* and *SAINT* (Clabbers *et al.*, 2018 ▸; Kabsch, 2010*b* ▸; Bruker, 2004 ▸) make it possible to describe complex experimental setups with an arbitrary rotation axis and a very flexible description of the detector geometry. The description of a diffraction experiment based on the rotation method requires only a small set of parameters:

(i) the direction and orientation of the rotation axis,
(ii) the oscillation width, *i.e.* the angle of rotation per recorded frame,
(iii) the detector distance (also known as the camera length),
(iv) the origin of the detector plane (several segments are possible) and
(v) the incident-beam direction.

Using such a setup, it was recently demonstrated that carrying out an electron diffraction experiment for chemical crystallography is very similar to carrying out an X-ray diffraction experiment, just with much smaller crystals (Gruene, Wennmacher *et al.*, 2018 ▸). In the present work, we describe the prototype setup of the electron diffractometer used in this study. At the core of the prototype diffractometer was an EIGER X 1M detector (DECTRIS Ltd), a hybrid pixel detector that is frequently used in X-ray crystallography but that is also suitable for detecting electrons (Tinti *et al.*, 2018 ▸). This hybrid pixel detector has a negligible readout time of 3 µs, which enables shutterless, continuous data collection (Broennimann *et al.*, 2006 ▸). It has a frame rate of up to 3 kHz, although in this study it was operated at 100 Hz. This enables fine-slicing of data (Pflugrath, 1999 ▸; Casanas *et al.*, 2016 ▸) at a fast rotation of the sample for instruments where the beam intensity cannot be arbitrarily reduced. The sensor layer of 450 µm makes it radiation-hard at electron energies of 200 keV and makes a beam stop unnecessary (Tinti *et al.*, 2018 ▸). The EIGER X 1M has a high count rate of 0.5 × 10^8^ s^−1^ mm^−2^, which was saturated only by the top 2–3 pixels of the direct beam and by none of the reflections. At 100 Hz, the dynamic range of 16 bit was well above all recorded intensities. The detector is noise-free, and only scattering inside the TEM added to the noise in the data. The prototype diffractometer also includes solutions for proper calibration of the instrument, *i.e.* the reliable determination of experimental parameters. This work may act as a reference for future instrument design, and aims at encouraging other research groups to determine chemical structures from submicrometre-sized crystals with existing TEMs before a fully integrated solution becomes available.

## 2. Methods and materials   

### 2.1. Instrumentation   

An FEI (now Thermo Fisher) Tecnai F30 transmission electron microscope at ScopeM, ETH Zurich was used as the basis for setting up the prototype diffractometer. The F30 was equipped with a Fischione Model 3000 HAADF-STEM detector. All data were collected at a beam energy of 200 keV, corresponding to an electron wavelength λ of 0.02508 Å. An EIGER X 1M detector (DECTRIS Ltd) was mounted inside a cylindrical vacuum chamber on-axis below the TEM camera chamber. Its lead shielding (Scherrer Metec AG, Zürich; 8 mm) was first evaluated using a Philips CM200-FEG microscope at C-CINA. Both setups were commissioned to comply with the EURATOM/2013/59 directive (European Union, 2013 ▸). The vacuum in the camera chamber reached 2 × 10^−6^ mbar after overnight pumping with the vacuum system of the respective TEM.

### 2.2. Calibration of rotation axis, detector distance and microscope magnification   

**Figure 1:**
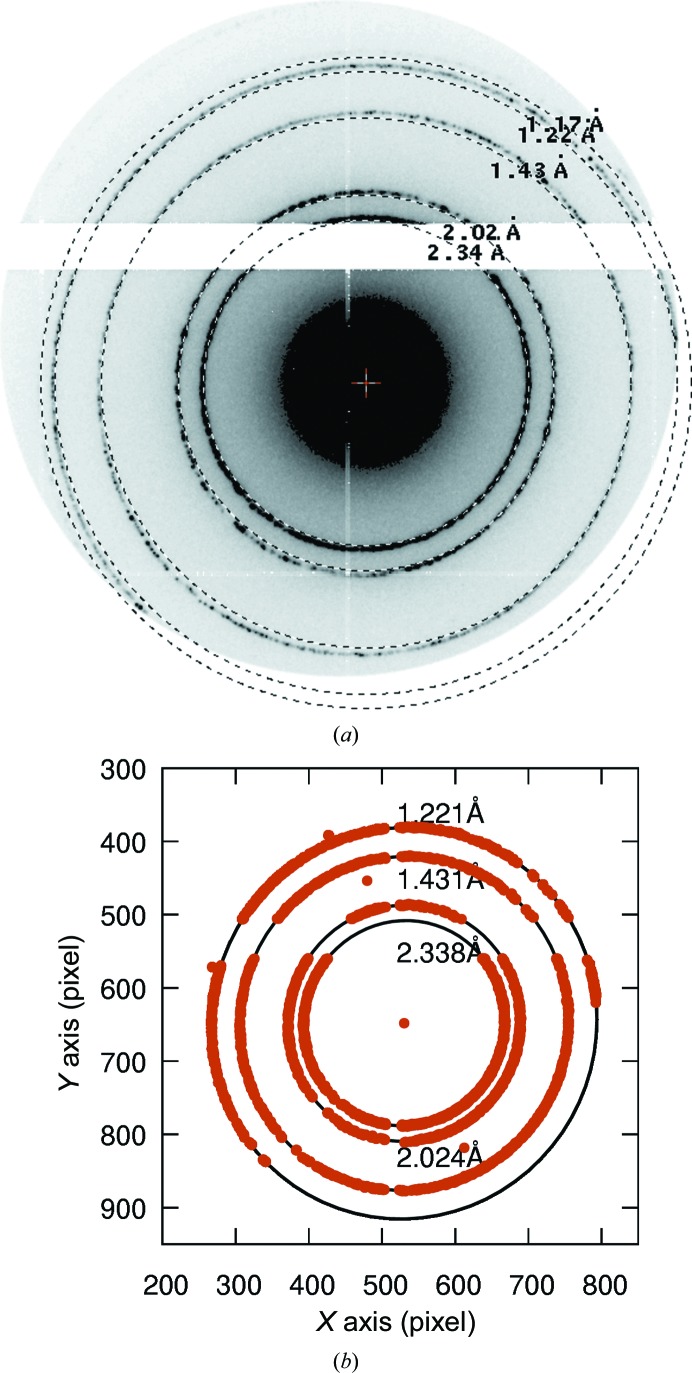
(*a*) Aluminium powder pattern recorded with oscillation of the rotation axis. Visible resolution rings are at 2.338, 2.024, 1.431 and 1.221 Å. (*b*) Fitting of strong reflections to ellipses. The elliptical distortions of the rings were determined as *A*/*B* = 1.0273 (2.338 Å), *A*/*B* = 1.0239 (2.024 Å), *A*/*B* = 1.0231 (1.431 Å) and *A*/*B* = 1.0231 (1.221 Å), where *A* and *B* are the major and minor axes, respectively. See also Clabbers *et al.* (2017 ▸).

**Figure 2:**
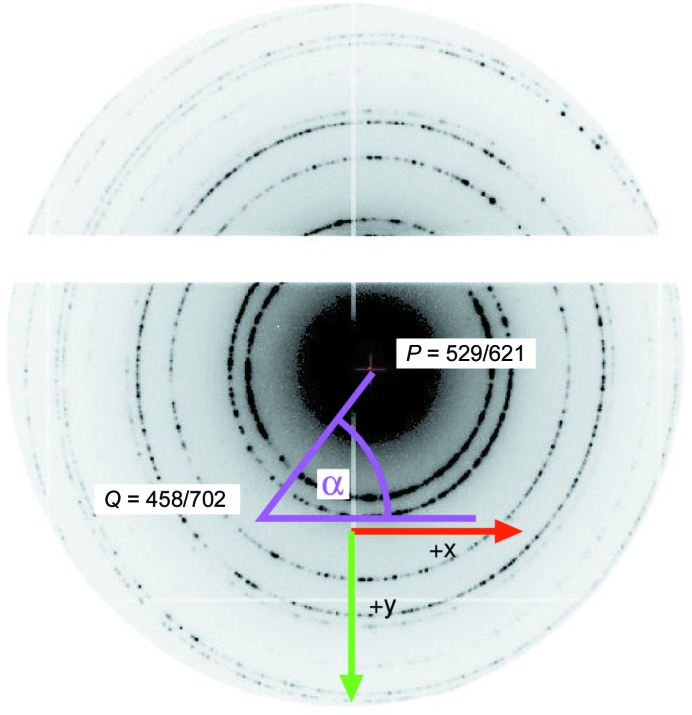
Summed images of an aluminium powder pattern recorded during oscillation of the grid. Reflections on or near the rotation axis stay in reflection conditions and thus have the strongest intensity on the ring. Likewise, a reflection-free segment on the rotation axis stays reflection-free during rotation. The minimum on the ring is easier to see than the maximum. Therefore, the rotation axis is determined by a line through the ring minimum and the direct-beam position.

The effective detector distance and the orientation of the rotation axis were determined with an aluminium powder standard (TedPella Inc.). The powder pattern was recorded with a frame rate of 100 Hz while it was oscillated by ±15° with the specimen-stage α-tilt wobbler. The peaks on the powder rings were found with the spot-finding procedure in *XDS.* The peaks were sorted according to the distance to the direct-beam position, and for each aluminium ring they were separated manually into individual files. The program *FITELLIPSE* (TG) fitted each set to an ellipse (Fig. 1 ▸). In this way it determined the ellipse centre and direction and the amplitude of both the major axis *A* and the minor axis *B*, as well as the effective detector distances based on either axis and the resolution of the corresponding powder ring. Alternatively, when the elliptical distortion is small, rings corresponding to the aluminium resolution can be fitted manually with the program *ADXV* by adapting the detector distance in the settings window. Only the pixel size of the EIGER X 1M detector, *i.e.* 75 µm, the wavelength (λ = 0.02508 Å) and direct-beam position are required. The main rings for aluminium have resolutions of 2.338, 2.024, 1.431, 1.221, 1.1690, 1.012, 0.9289, 0.9055 and 0.8266 Å. In order to determine the rotation axis, the frames of the aluminium powder pattern of at least one oscillation period were summed. The rotation axis runs approximately along the line between the point of minimum intensity on the ring and the direct beam (Fig. 2 ▸). The maximum intensity could be used instead of the minimum, but the minimum is easier to spot by eye. The magnification on the EIGER X 1M detector in imaging mode was determined from a standard gold cross-grating with 2160 lines per millimetre, *i.e.* 463 nm per box width (Agar Scientific S106).

### 2.3. Oscillation width   

The α-tilt stage rotation was set to 10% or 20% of its full speed. To determine the oscillation width, we recorded the φ angle (known as the α angle in TEM terminology) at 0.5 s intervals during the measurements. The script is described in Appendix *A* [App appa]. The resulting plot was fitted to a straight line φ(*t*) = 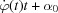 with *gnuplot*. The oscillation width Δφ per frame was calculated from the detector readout frequency ν as Δφ = 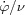.

**Figure 3:**
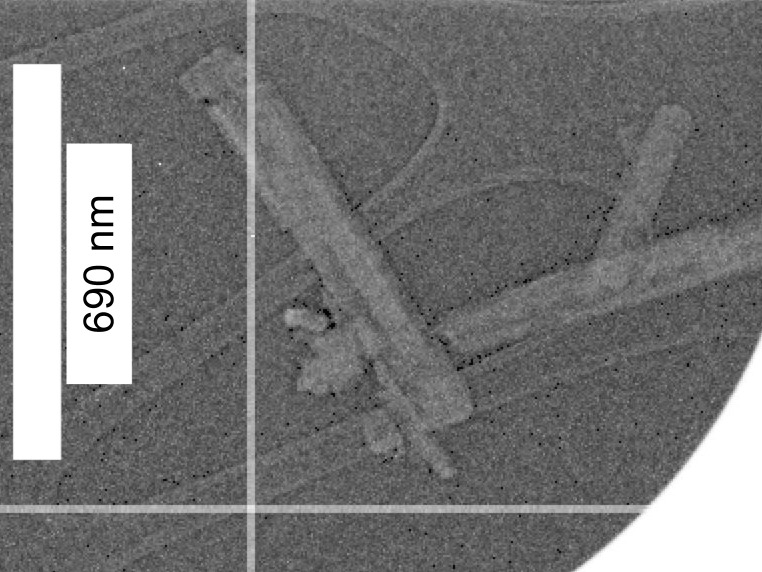
Two bar-shaped crystals with smaller satellite crystals. The image was recorded in the TEM using the EIGER X 1M detector with a dose of 10^−3^ e^−^ Å^−2^.

Some microscopes, such as the Philips CM200-FEG, can only set the rotation rate with a continuous turn button or a pressure-sensitive button without a scale. With such microscopes, the oscillation width varies for every data set. In this case, the rotation of the stage was recorded with a camera and screenshots were taken with the program *MPV* (https://mpv.io) using the command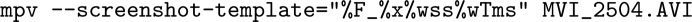 Fig. 3 ▸ illustrates how to determine the oscillation width from two screenshots.

### 2.4. Sample search and estimate of electron dose   

Samples were searched in HAADF-STEM mode or with low magnification and low dose in imaging mode on the EIGER X 1M detector visualized with *ALBULA* (DECTRIS Ltd). When the sample search was carried out using the HAADF-STEM detector of the FEI F30 microscope, we used spot size 9. At spot size 8, the reading of the screen current is 50 pA. The instrument does not report lower values. The current is halved per increment in spot size, so we assume the current to be 25 pA at spot size 9. The dwell time was 4 µs per pixel. At an image size of 512 × 512 pixels, and 1500× magnification, the pixel size corresponds to 194 nm on the sample. The dose per image is thus calculated as
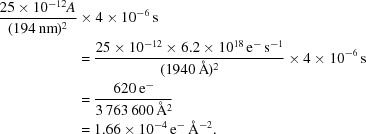
Note that the calculations depend on the square of the magnification.

**Figure 4:**
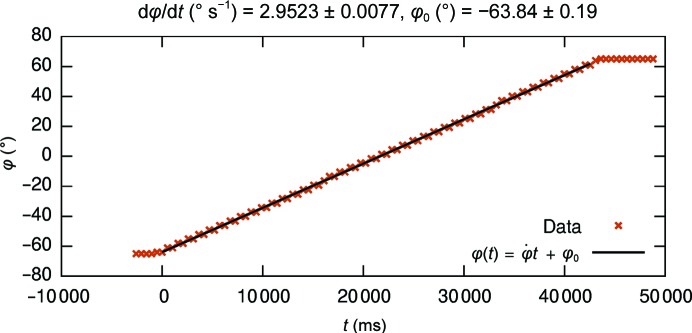
Plot and fit of the rotation angle φ during data collection. In the script that records the stage angle, the absolute time is recorded just before (*t*_before_) and just after (*t*_after_) the call for the stage angle, so that the error in the *x* coordinate is 0.5(*t*_after_ − *t*_before_). Error in *y* is not taken into account.

For comparison between STEM mode and imaging with the EIGER X 1M, Fig. 4 ▸ shows a single frame of a crystal recorded at 0.01 e^−^Å^−2^ s^−1^ at 100 Hz, *i.e.* a dose of 10^−3^ e^−^ Å^−2^. While the contrast is not as good as with HAADF-STEM, it is sufficient to locate crystals. In combination with an automated search procedure (Smeets *et al.*, 2018 ▸), samples can be searched at very low dose directly with an EIGER X 1M detector, for example when an HAADF-STEM detector is not available.

### 2.5. Alignment of rotation axis (eucentric height)   

The alignment of the crystal with the rotation axis of the stage is also called ‘setting the eucentric height’ in electron microscopy. As with X-rays, the crystal is aligned when it does not change position in the *xy* plane upon rotation. In electron crystallography, where the electron wave is only planar at a specific position depending on the electron optics, the sample should simultaneously be in focus. In imaging this corresponds to minimum contrast of the image, which is a fast way to align the rotation axis. Mechanical alignment was performed by ‘wobbling’ the stage, *i.e.* an oscillation between φ = −30° and φ = 30° while changing the *z* height until the sample stayed fixed (Zuo & Spence, 2017 ▸). Radiation-sensitive samples were moved out of the beam parallel to the *xy* plane during alignment. Data collection started directly after moving the sample back without repeating the alignment procedure. The rotation axis was always moved beyond the starting angle and from there to the starting angle before data collection started. This reduces the effect of a backlash in the rotation mechanism. A quasi-parallel beam was reached by choosing the smallest available condenser aperture, 30 µm on the F30, combined with a small spot size to reduce the dose on the sample (Valery *et al.*, 2017 ▸). The beam diameter was about 1.5 µm for the F30, as determined by imaging.

## 3. Results   

The prototype electron diffractometer, including its calibration setup, was validated using diffraction data collected from several crystals of ZSM-5, a zeolite with the MFI framework (Baerlocher & McCusker, 2016 ▸). The performance of the diffractometer with respect to chemical crystallography has been shown by the determination of several structures (Gruene, Wennmacher *et al.*, 2018 ▸). The data were analysed with *XDS* (Kabsch, 2010*b* ▸). All experimental parameters are instrument-dependent. They can and should be calibrated before the actual experiment. Most instruments are sufficiently stable so that calibration is only necessary at regular (for example weekly) intervals and not for every data collection.

### 3.1. Rotation axis   

The initial rotation axis **R**_init_ was determined manually from the sum of images from an oscillating aluminium powder standard. The rotation axis runs through the direct-beam position and the two intensity minima of each powder ring (Fig. 2 ▸),
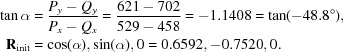
The manual determination of the rotation axis was accurate within about 5°. This was within the radius of convergence of the indexing procedure in *XDS* (Kabsch, 2010*a* ▸). The twofold ambiguity was clearly resolved from the standard deviation of the spot and spindle positions reported in the log file of the IDXREF step and the CORRECT step of *XDS*. The rotation axis was determined once per setting of the projector lens, which determines the orientation of the back focal plane of the objective lens, and thus the orientation of the diffraction images. The axis can be refined with diffraction data from well diffracting crystals such as ZSM-5.

### 3.2. Detector distance   

The electron optics in a TEM are subject to aberrations. These lead to distortions of the diffraction pattern (Capitani *et al.*, 2006 ▸). *XDS* has per-pixel mechanisms to correct for distortions in the detector plane with subpixel precision. Correcting for the distortion leads to slightly improved cell accuracy (Capitani *et al.*, 2006 ▸; Ångström *et al.*, 2018 ▸). Better prediction of spot positions leads to better background estimates and thus to more reliable intensities and better *I*/σ(*I*) values. Fig. 1 ▸ shows the elliptical distortion of an aluminium powder pattern. In this case, the ellipticity *e* = *A*/*B* − 1 with major axis *A* and minor axis *B* is about 2.3%. This small distortion did not prevent structure solution, and we did not apply corrections. The detector distance can be calculated from either the major or the minor axis. The ambiguity can be resolved with a sample with known unit-cell parameters. The distance calibration should be repeated regularly, as the level of distortion varies with lens settings.

### 3.3. Oscillation width   

**Figure 5:**
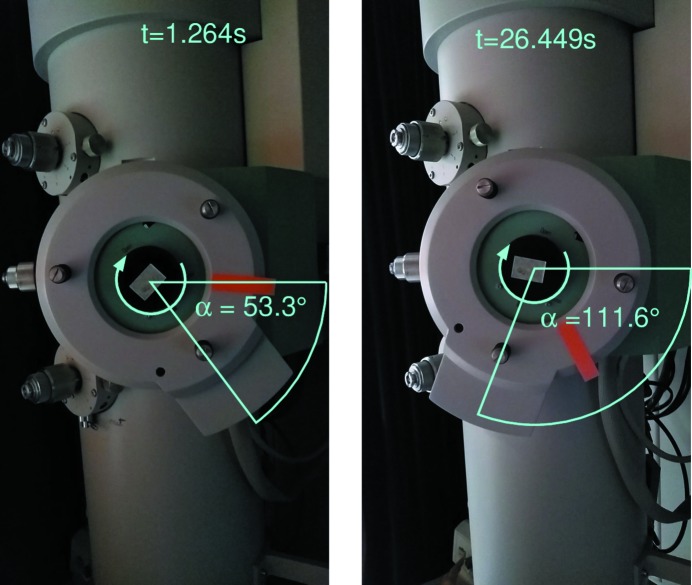
Determination of the oscillation width from movie screenshots in the absence of a digital readout. Δφ = (Δα/Δ*t*) × ν(EIGER) = (111.6° − 53.3°)/ (26.45 s − 1.26 s) × 100 Hz = 0.02315° per frame.

On the Tecnai F30 TEM used in this study, the rotation rate was read out during data collection with a scripting language available on the F30 (Appendix *A* [App appa]). Integration programs assume a constant oscillation width, *i.e.* the angular difference between adjacent frames. It is the only experimental parameter of those listed in Section 1 that is not refined. The oscillation width is the ratio 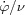 between the rotation rate 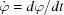 and the detector readout frequency ν. Hence, a detector with precise and accurate timing electronics contributes to the reliability of the oscillation width. An example graph for the rotation rate is shown in Fig. 4 ▸. Although the angle value was only recorded every 0.5 s, the script reports identical angles for every two readout steps. This was consistent for each recording of our experiments. In addition to this observation, the oscillation width shows a large variation between data sets (Table 1 ▸). We suspect a non-constant oscillation rate to be the reason, such as a faster rotation when a rotationally nonsymmetric weight moves down and a slower rotation when a rotationally nonsymmetric weight moves up (*cf.* Fig. 5 ▸). For a production diffractometer, the sensitive measurement of the rotation rate as described in Gemmi *et al.* (2015 ▸) may be advisable. We generally assumed 2.95° s^−1^ for our experiments. Given that rotationary motors with higher precision and accuracy are available (Schneider *et al.*, 2014 ▸; Waltersperger *et al.*, 2015 ▸), we consider the goniometer to be the most important piece of hardware that should be improved in future instruments. Instruments newer than the Tecnai F30 or the CM200-FEG may show greater stability of the rotation rate, although also other groups have reported poor precision for this parameter (Hattne *et al.*, 2015 ▸).

### 3.4. Incident-beam direction and origin of the detector plane   

As the direction of the incident beam is one of the refined parameters, and since it does not deviate greatly from orthogonality to the detector plane, it can initially be set to (0, 0, 1) in the laboratory coordinate system defined in *XDS*. Likewise, the origin of the detector coordinate system can be set to the position of the direct beam on the detector plane. The EIGER detector has a Si sensor layer with 450 µm thickness. Electrons at 200 keV cannot penetrate this layer, so there is no need for a beam stop. The direct-beam position is therefore read directly from the diffraction image. It can in principle vary between data sets, but with good instrument settings it was stable within each data set and across different data sets.

### 3.5. Reflecting range and beam divergence   

The mosaicity of the crystal affects the reflecting range and is strongly entangled with the beam divergence. Using the Kabsch projection (Kabsch, 1988 ▸), the spot shape can be modelled as a two-parameter Gaussian based on the reflecting range and the beam divergence (Kabsch, 2010*a* ▸). The reflecting range is closely related to the crystal mosaicity and thus is a sample-specific parameter. *XDS* suggests these values in the log file of the INTEGRATE step. This procedure, however, does not have a large radius of convergence (W. Kabsch, private communication). Without initial starting values, unrealistically large values for the reflecting range were suggested for most of our data sets, often of several degrees. Starting values can be estimated with a diffraction-image viewer such as *ADXV* (Arvai, 2018 ▸). The reflecting range corresponds to the angle value for which reflections perpendicular to the rotation axis are visible on the diffraction frames. The beam divergence of the F30 reported by *XDS* was about 0.07–0.08°. This is about one order of magnitude smaller than for X-ray sources. We observe that a proper setting of these values improves the *I*/σ(*I*) value. Too small values for the reflecting range exclude part of the intensity, leading to poor CC_1/2_ values, while too large values increase the background region integrated as intensity, thus reducing the *I*/σ(*I*) value by the inclusion of noise.

The rotation rate of 2.95° s^−1^ resulted in about 1 min per data set. For daily structure determination at an X-ray facility these are competitive numbers, and match recently developed automated procedures in 3D electron diffraction (Smeets *et al.*, 2018 ▸). Calibration of the experimental parameters makes it possible to prepare a template input file for the integration program of choice. Data processing can thus start on-site while the operator collects data from several crystals.

## 4. Conclusions   

We mounted the EIGER X 1M hybrid pixel detector to a transmission electron microscope. Processing of diffraction data collected using the rotation method requires knowledge of only a small set of experimental parameters. We describe how to determine those experimental parameters and how data processing can be started in parallel to data collection. Hence, a prototype electron diffractometer has become available for any X-ray facility for chemical crystallography. Since many of the properties of high-end TEMs are not necessary for diffraction studies, the investment for a TEM suitable for 3D electron diffraction is of the same order of magnitude as for a modern X-ray diffractometer.

This prototype electron diffractometer is suitable for production work. However, a fully integrated electron diffractometer would be desirable. Table 2 ▸ summarizes the features that an electron diffractometer should have. All of these features are feasible considering state-of-the-art engineering. In addition, a horizontal layout of the instrument (Vainshtein, 1964 ▸) could feature a vertical sample holder that is inserted from top to bottom. If such a sample holder were rotationally symmetric about the vertical axis, this system would have several important advantages. Owing to the symmetry, rotation does not change the leverage point of the holder. This avoids slippage of the sample during rotation and stabilizes the oscillation width throughout the rotation range. With a horizontal rotation axis, the pivot point is different between room-temperature holders and cryo-holders with a reservoir for liquid nitrogen. A vertical axis enables the same calibration for room-temperature holders and cryo-holders. In combination with high-precision rotation systems (Waltersperger *et al.*, 2015 ▸), a vertical goniometer provides a sphere of confusion of better than 100 nm (Schneider *et al.*, 2014 ▸). In addition, the detector surface would be protected from loose particles. Finally, continuous rotation would be possible. Except for the horizontal layout, our results demonstrate that these features are accessible with the necessary accuracy and that it is only a matter of modern engineering to produce a proper electron diffractometer. Before such an instrument becomes available, electron crystal structure determination can be carried out with current instrumentation that is affordable to many research institutes. Our work thus describes the essential guidelines for the design of a dedicated electron diffractometer: the missing link to make electron crystallography a fruitful extension to X-ray crystallography in chemical crystallography (Simancas *et al.*, 2016 ▸).

## Figures and Tables

**Figure 6:**
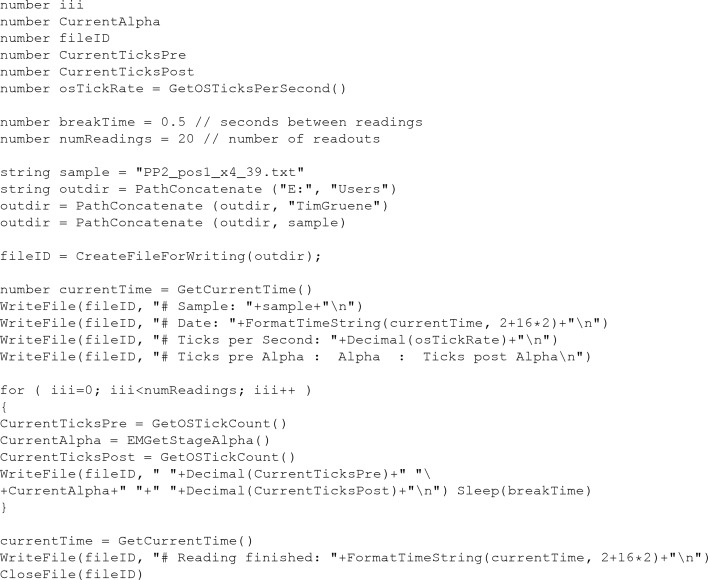
Script for recording the rotation angle φ.

**Figure 7:**
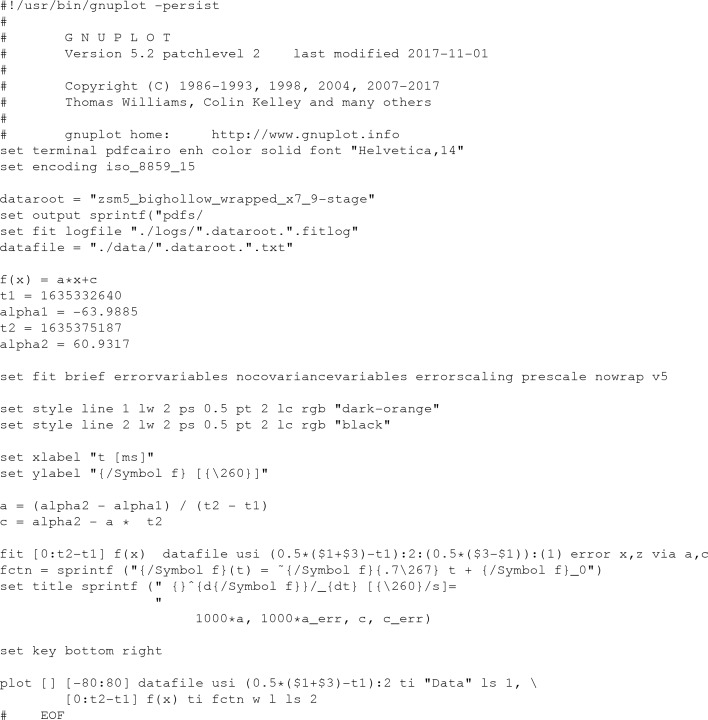
Script for data fitting to a linear function.

**Table 1:** Selection of rotation rates of the goniometer determined by fitting a straight line to the readout values of the instrument angles (Appendix *A* [App appa]) ID corresponds to the ID of data sets available at Zenodo (https://doi.org/10.5281/zenodo.1297083). A negative rate corresponds to the reverse rotation direction.

ID	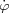 ± 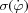 (° s^−1^)	φ_total_ (°)
gstad_x1_6	2.9333 ± 0.0482	35.7
gstad_x2_7	2.9104 ± 0.0271	53.6
gstad_x4_9	2.9853 ± 0.0269	56.6
gstad_x5_10	2.8863 ± 0.0311	47.5
IR7b_2_pos14_x1_10	3.0301 ± 0.1321	20.9
IR7b_2_pos14_x1_11	2.9734 ± 0.1171	23.6
IR7b_2_pos14_x1_21	−2.8640 ± 0.0919	−23.8
IR7b_2_pos14_x1_22	−2.9116 ± 0.1016	−20.8
IR7b_2_pos14_x1_23	−2.9048 ± 0.1016	−20.8
IR7b_2_pos14_x1_24	−2.8718 ± 0.1026	−20.9
zsm5_bighol_NFi2_x10_9	2.9569 ± 0.0088	118.6
zsm5_bighol_NFi2_x11_11	2.9538 ± 0.0080	122.0
zsm5_bighol_NFi2_x5_9	2.9510 ± 0.0080	125.1
zsm5_bighol_NFi2_x6_11	2.9530 ± 0.0087	116.1
zsm5_bighol_NFi2_x8_5	2.9593 ± 0.0090	113.0
zsm5_bighol_NFi2_x9_7	2.9525 ± 0.0078	125.0

**Table 2:** List of desirable components for an electron diffractometer

Item	Description	Reference(s)
Köhler illumination	Ensures parallel beam at sample	Benner & Probst (1994 ▸)
STEM	Low dose of sample imaging; beam position should be maintained between STEM mode and diffraction mode	
High-precision goniometer	Full sample rotation enables two-click centring; modern mechanics with 100 nm precision enable reduced beam size	Schneider *et al.* (2014 ▸), Waltersperger *et al.* (2015 ▸)
Parameter readout	Experimental parameters required for data processed transferred as metadata	See main text
Energy filter	Noise reduction	Yonekura *et al.*, 2002 ▸)
Horizontal layout	Improved goniometer (precision)	Vainshtein (1964 ▸)

## Appendix A Determination of the oscillation width

### Recording the rotation angle φ

This script is written for *DigitalMicrograph* (Gatan Inc.). The filename and directory path are created with the command PathConcatenate. In the example in Fig. 6 ▸, the log file is written to E:\Users\TimGruene\PP2_pos1_x4_39.txt.^1^ The script was started before and stopped after the collection of the data set to ensure that the entire rotation is recorded. Note that access to the instrument parameters does not depend on the presence of a third-party tool such as *DigitalMicrograph* (Cichocka *et al.*, 2018 ▸).

### A2. Data fitting to a linear function

As shown in Fig 4 ▸, the data recorded with the script listed in Fig. 6 ▸ show two constant regions when the rotation axis is not moving and a region with a linear increase or decrease corresponding to a rotation clockwise or anticlockwise, respectively. The nonconstant segment was fitted to a linear function  using the script in Fig. 7 ▸. The string for dataroot sets the file names for input data, output PDF and output log file. The start and end points, *t*_1_, α_1_, *t*_2_, α_2_, must be inserted manually. The title of the plot file shows the rotation rate  (° s^−1^). The oscillation width Δφ (° per frame) required for data processing can be calculated *via* the detector frame rate ν (Hz) as .
